# Towards Accurate Reference Values for Heart Rate and Speed Zones by Aerobic Fitness and Sex in Long-Distance Runners

**DOI:** 10.3390/sports14010029

**Published:** 2026-01-07

**Authors:** Jonathan Esteve-Lanao, Sergio Sellés-Pérez, Héctor Arévalo-Chico, Roberto Cejuela

**Affiliations:** 1 All In Your Mind Training System Ciudad de México, Mexico City 06140, Mexico; jonathan.esteve@allinyourmind.es; 2 Biking Performance Hub, BPH, Mexico City 11550, Mexico; 3 Physical Education and Sports, Faculty of Education, University of Alicante, 03690 San Vicente del Raspeig, Spain; sergio.selles@ua.es (S.S.-P.); roberto.cejuela@ua.es (R.C.)

**Keywords:** endurance training, performance analysis, heart rate, VT1, VT2, running speed, oxygen consumption

## Abstract

Background: This study aimed to provide reference values for estimating training intensities in long-distance runners based on progressive incremental tests, considering differences related to sex and performance level. Methods: A total of 1411 endurance-trained runners (819 men and 592 women) completed a standardized treadmill protocol with gas exchange analysis to determine ventilatory thresholds and peak oxygen consumption (VO_2peak_). Heart rate (HR) and running speed at each threshold were expressed relative to their peak values. Results: HR at second ventilatory threshold (VT2) occurred at 93.5 ± 2.5% of HR peak, and HR at first ventilatory threshold at 85.1 ± 4.6%. The relative running speeds at VT2 and VT1 corresponded to 87.6 ± 3.9% and 73.9 ± 5.5% of the speed at VO_2peak_, respectively. In men, beginners exhibited higher relative HR and VO_2_ values at the ventilatory thresholds than elite runners. In contrast, women displayed higher and more stable relative values across performance levels. Conclusions: These findings establish precise, evidence-based reference ranges derived from a large cohort of runners and highlight the need to consider sex and performance level when estimating exercise intensities. Individualized physiological assessment remains essential for accurate training prescription and performance optimization.

## 1. Introduction

Exercise intensity is one of the key external load variables used to modulate the exercise dose administered to an athlete, together with exercise type, duration, and recovery [1]. Having effective strategies to control exercise intensity of each session is essential for the proper execution of the training plan and the enhancement of athletic performance [2]. Several variables can help determine the actual toughness of exercise and the level of physiological demand it imposes on the athlete. In the case of endurance runners, oxygen consumption and blood lactate concentration are considered the most valid and commonly used indicators in scientific research [3]. However, during regular training sessions, more practical measures such as running speed, rate of perceived exertion, and heart rate (HR) are typically preferred [4]. Associating these variables with specific physiological milestones can facilitate targeted work on the physiological determinants of performance at any given moment, especially those related to maximal oxygen consumption (VO_2max_) and the ventilatory thresholds, ventilatory threshold 1 (VT1) and ventilatory threshold 2 (VT2) [5]. In this context, training zones provide a useful framework for defining specific intensity ranges—commonly based on running speed or HR—within which the desired training stimulus can be precisely applied [2]. To achieve this, it is essential that training zone calculations are based on direct testing, preferably involving gas exchange analysis or blood lactate measurement, using methodologies validated by the scientific literature [6].

However, it is often difficult for athletes and coaches to access the equipment and infrastructure required to perform these tests, which forces them to estimate training zones indirectly. Some of the most used methods are based on single-effort prediction trials [7], maximal HR, or the HR corresponding to the metabolic thresholds, among others [2]. From these reference values, percentage-based estimations can be applied to predict HR or speed at other physiological landmarks or to prescribe exercise intensities [8].

Several studies have demonstrated the accuracy of fixed-intensity anchors (e.g., specific percentages of maximal HR, VO_2max_, or maximal aerobic speed) for estimating VT2 and VT1 in runners [9,10,11]. Nevertheless, other studies suggest that these percentage values can vary considerably between individuals, with factors such as sex and training status potentially influencing these estimations [12,13,14]. Specifically, sex is an important factor to consider when attempting to individualize training zones. Traditionally, women’s training programs have been established following the same standards as those of men, despite the fact that physiological responses to exercise differ between the sexes [15]. Considering sex-specific responses is therefore essential not only for optimizing training outcomes but also for accurately interpreting performance and physiological testing data.

In addition, it should be noted that in progressive incremental tests to exhaustion, lower-level athletes may not always reach their VO_2max_, possibly due to a lower physiological resilience or tolerance to high-intensity effort [16,17]. This limitation can introduce bias when defining maximal reference values, particularly when these are later used to estimate training zones or metabolic thresholds indirectly.

On the other hand, in recent years, there has been a considerable increase in the use of training monitoring platforms and wearable devices among both recreational and trained runners [18]. These platforms often include automated tools for calculating training zones; however, these zones are frequently too generic and fail to account for individual variables that can significantly influence their accuracy and applicability [18]. Therefore, having reference values derived from direct measurements in a large cohort of runners—including various performance levels and both sexes—may help coaches and athletes with limited access to laboratory testing to more accurately determine their training zones using progressive exercise tests performed to volitional exhaustion.

Thus, the aim of the present study was to analyze and present absolute and relative data for running speed, HR, and oxygen consumption (VO_2_) with respect to their maximal values at VT1 and VT2 in trained long-distance runners of different performance levels (from recreational to elite) and of both sexes, obtained from a progressive incremental performance test performed until exhaustion. In this way, percentage reference values can be established for the different metabolic milestones.

The hypotheses proposed based on previous scientific literature were as follows: (1) Female runners will present higher relative HR values (as a percentage of maximum) at VT1 and VT2 compared with male runners. (2) Female runners will present higher relative running speed values (as a percentage of maximal speed reached at the test) at VT1 and VT2 compared with male runners. (3) The lower ability to sustain high-intensity efforts in lower-performance athletes will result in higher relative running speed and HR values (as a percentage of maximal aerobic speed and HR peak value) at VT1 and VT2 compared with higher-performance athletes.

## 2. Materials and Methods

### 2.1. Participants

A total of 1411 athletes participated in the study, comprising 819 men (173.8 ± 7.4 cm, 74.4 ± 10.7 kg, and 37.1 ± 12.4 years) and 592 women (162.3 ± 6.6 cm, 60.0 ± 9.5 kg, and 37.0 ± 11.8 years). All participants were habitual runners with a minimum of one year of experience in structured endurance training. Participants were required to perform a minimum of three running training sessions per week. The athletes were recruited from two training groups specifically dedicated to distance running training and competition. The athletes voluntarily completed the performance tests between 2021 and 2025. All tests were conducted between the 2nd and 5th week of training in each season. The test was conducted after a period of reduced training between competitive seasons (2 to 3 weeks), during the early preparatory phase.

All participants provided their informed consent prior to the use of their data in this research. The study procedures were reviewed and approved by the Ethics Committee of the University of Alicante (protocol code: A-2017-04-11 expedient). All data collection procedures adhered to the principles outlined in the Declaration of Helsinki.

### 2.2. Procedures

The running performance test was performed on a treadmill (Woodway PRO XL, Waukesha, WI, USA). All tests were conducted in the morning (08:00–11:00 h). Participants were instructed to avoid strenuous exercise for 24 h prior to testing. The protocol started at a 1% gradient, with increments of 0.3 km·h^−1^ every 30 s until volitional exhaustion [19]. The initial speed was individually determined to ensure that the total test duration ranged between 10 and 15 min and did not exceed 26 min [20].

A portable gas exchange analyzer (Cosmed K5, Rome, Italy) was used to determine ventilatory thresholds and the peak value of VO_2_ (VO_2peak_). The recorded respiratory variables included VO_2_, pulmonary ventilation (VE), ventilatory equivalents for oxygen (VE/VO_2_) and carbon dioxide (VE/VCO_2_), and end-tidal partial pressures of oxygen (PETO_2_) and carbon dioxide (PETCO_2_).

VO_2peak_ was defined as the highest 1-min average VO_2_ value obtained during the test. VT1 and VT2 were determined according to the criteria described by Davis et al. [21]. VT1 was identified by a systematic increase in both VE/VO_2_ and PETO_2_ without a concomitant rise in VE/VCO_2_, whereas VT2 was defined by a simultaneous increase in VE/VO_2_ and VE/VCO_2_ accompanied by a decline in PETCO_2_. Both VT1 and VT2 were independently assessed by three experienced evaluators. Other data were collected during the test including voluntary exhaustion of the participant, HR ≥ theoretical HRmax (calculated as 208–0.7·age [22]). The corresponding running speed at VO_2peak_ (SVO_2peak_) and at VT1 (SVT1) and VT2 (SVT2) was recorded.

Additionally, running economy (RE) was assessed following the warm-up. Participants performed a 6-min constant-speed run at an intensity corresponding to their estimated VT1, determined from recent training sessions. VO_2_ was continuously measured throughout the test, and the mean VO_2_ from the final 3 min was used for the calculation. Running economy, expressed as mL·kg^−1^·km^−1^, was calculated using the equation: (60 divided by the running speed in km·h^−1^) multiplied by the average VO_2_ value in mL·kg^−1^·min^−1^. Furthermore, the relative VO_2_ at VT2 and VT1 was calculated as a percentage of VO_2max_. HR was continuously monitored throughout the test using a telemetry-based system (Polar Verity Sense, Polar Electro, Kempele, Finland). The peak heart rate value (HR_peak_) was defined as the highest value recorded during the test. In addition, HR values were associated with the HR corresponding to VT2 (HRVT2) and VT1 (HRVT1). The relative HR as a percentage of the HR_peak_ at VT2 and VT1 was calculated. Also, the relative HR as a percentage of HRVT2 at HR_peak_ and VT1 was calculated. Finally, the relative running speeds at VT1 and VT2 were calculated as percentages of both SVO_2peak_ and the speed corresponding to VT2.

For the subsequent analysis, athletes were categorized according to their performance level based on their VO_2peak_ values. The classification criteria proposed by Barnes and Kilding [23] were used to define the performance groups as follows: Very high aerobic fitness (elite) men > 75.4 mL·kg^−1^·min^−1^; High aerobic fitness men 70.8–75.4 mL·kg^−1^·min^−1^; moderately aerobic fitness men 62.2–70.0 mL·kg^−1^·min^−1^; low to moderate aerobic fitness men 54.2–62.2 mL·kg^−1^·min^−1^; and low aerobic fitness (beginners) men < 54.2 mL·kg^−1^·min^−1^. For women, very high aerobic fitness (elite) women > 66.2 mL·kg^−1^·min^−1^; High aerobic fitness women 61.7–66.2 mL·kg^−1^·min^−1^; moderately aerobic fitness women 55.8–61.7 mL·kg^−1^·min^−1^; low to moderate aerobic fitness women 49.7–55.8 mL·kg^−1^·min^−1^; and low aerobic fitness (beginners) women < 49.7 mL·kg^−1^·min^−1^.

### 2.3. Data Analysis

A descriptive analysis was conducted using the mean and standard deviation (SD) of all variables under study. An independent samples t-test was performed to analyse potential differences in the recorded variables according to performance level and sex. For comparisons involving the very high aerobic fitness males, very high aerobic fitness females, and high aerobic fitness female groups, the nonparametric Mann–Whitney U test was used to detect statistical differences between variables. Pearson’s bivariate correlation coefficient was used to determine the existence of any inter-relationships between the measured variables. Effect size was calculated using Cohen’s d [24] interpreted as follows: trivial (0–|0.2|), small (|0.2|–|0.4|), moderate (|0.4|–|0.8|), and large (>0.8) [25]. Prior to the analyses, the normality of the data was verified using the Kolmogorov–Smirnov (KS) test, confirming a normal distribution. Homogeneity of variances was assessed with Levene’s test. All statistical analyses were performed using the Statistical Package for the Social Sciences (SPSS, version 22.0; IBM Corp., Chicago, IL, USA). Statistical significance was set at *p* < 0.05.

## 3. Results

Table 1 presents the physiological data obtained from the progressive exercise test. Higher running speeds at VT1 and VT2 and at VO_2peak_ were observed in higher-level athletes for both men and women. Better running RE values were recorded in the higher-level runners of both sexes. Additionally, lower percentages of VO_2_ relative to VO_2peak_ at VT1 and VT2 were evident in higher-level male athletes, whereas no such trend was observed in female athletes. The percentage of participants reaching the theoretical maximal HR were higher in athletes of greater performance level. Overall, these differences were more pronounced in men than in women. In addition, the number of years of training and competitive experience can be observed in the dataset and descriptively shows that the groups with higher physiological performance presented a greater number of years of both training and competitive experience.

Table 2 presents the relative HR and running speed values obtained from the performance test, along with comparisons between male and female athletes across different performance levels. Considering the entire sample, the HRVT2 corresponded to 93.5 ± 2.5% of HR_peak,_ while HRVT1 represented 85.1 ± 4.6% of HR_peak_. Conversely, when taking HRVT2 as a reference point, HR_peak_ corresponded to 107.0 ± 2.9%, and HRVT1 to 90.9 ± 3.7%. The relative running speed at VT2 with respect to SVO_2peak_ was 87.6 ± 3.9%, while the relative speed at VT1 was 73.9 ± 5.5%. When using the running speed at VT2 as the reference, the relative SVO_2peak_ corresponded to 114.4 ± 5.3%, and the relative speed at VT1 was 84.4 ± 4.7%.

**Figure 1:**
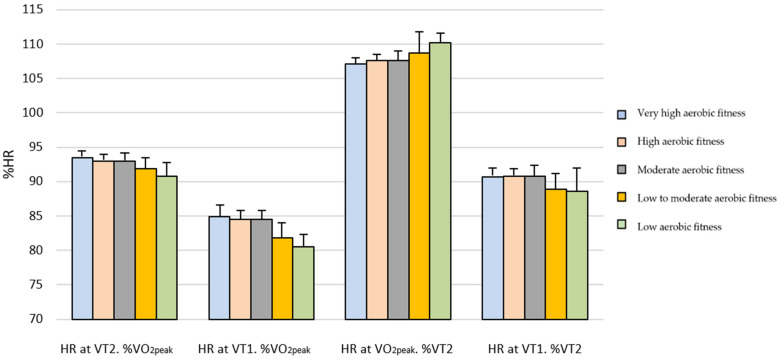
Relative heart rate according to performance level in male athletes.HR at VT2. %VO_2peak_ = Relative heart rate values at second ventilatory threshold as percentage of the peak heart rate; HR at VT1. %VO_2peak_ = Relative heart rate values at first ventilatory threshold as percentage of the peak heart rate; HR at VO2peak. %VT2 = Relative heart rate values at the peak value of heart rate as percentage of the heart rate at second ventilatory threshold; HR at VT1. %VT2 = Relative heart rate values at the first ventilatory threshold as percentage of the heart rate at second ventilatory threshold.

**Figure 2:**
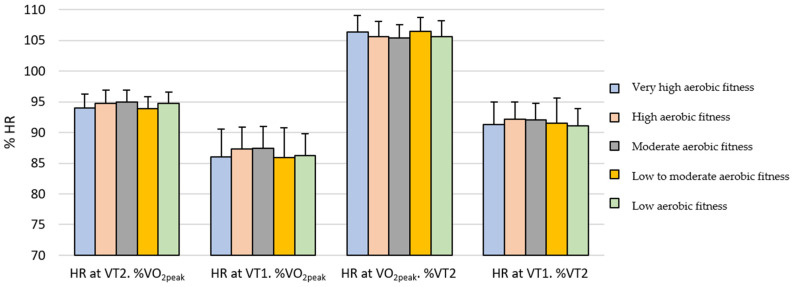
Relative heart rate according to performance level in female athletes. HR at VT2. %VO_2peak_ = Relative heart rate values at second ventilatory threshold as percentage of the peak heart rate; HR at VT1. %VO_2peak_ = Relative heart rate values at first ventilatory threshold as percentage of the peak heart rate; HR at VO_2peak_. %VT2 = Relative heart rate values at the peak value of heart rate as percentage of the heart rate at second ventilatory threshold; HR at VT1. %VT2 = Relative heart rate values at the first ventilatory threshold as percentage of the heart rate at second ventilatory threshold.

On the other hand, Table 2 shows that women exhibited significantly higher relative HR values, expressed as a percentage of HR_peak_, at VT1 and VT2, as well as higher relative HR values expressed as a percentage of VT2 at VT1, and lower values at HRpeak. Regarding relative running speed expressed as a percentage of SVO_2peak_, significantly higher percentages were observed at VT1 and VT2 in athletes with low aerobic fitness, low-to-moderate aerobic fitness, and moderate aerobic fitness, whereas no significant differences were found in the high and very high aerobic fitness groups. These differences can be visually observed in Figure 1 and Figure 2 for men and women, respectively.

**Figure 3:**
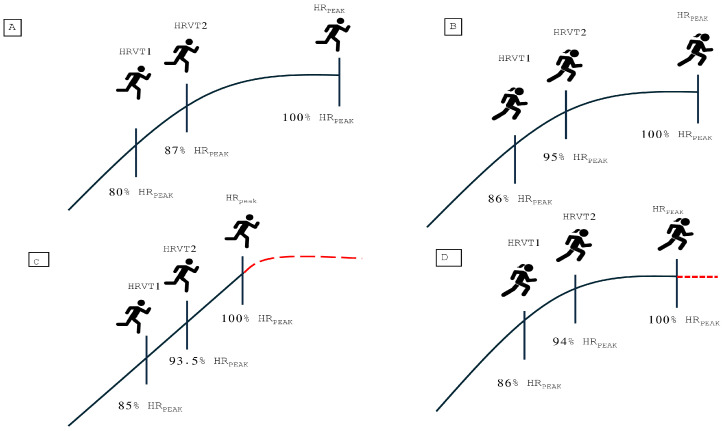
Absolute and relative peak heart rate values in very high aerobic fitness males (**A**), very high aerobic fitness females (**B**), low aerobic fitness males (**C**), and low aerobic fitness females (**D**) during a progressive exercise test.

Table 3 and Table 4 present the *p* values and effect sizes, measured using Cohen’s d, between performance-level groups in men and women for relative HR values expressed as a percentage of HR_peak_ at VT1 and VT2, respectively. In men, the largest differences were observed between groups with the greatest performance gap (e.g., between elite and beginner athletes) for both VT1 and VT2. In contrast, this trend was not evident in women, where few statistically significant differences were found between groups. Figure 3 shows an explanatory diagram of the absolute and relative heart rate values at different levels of aerobic performance.

From the correlation analysis, the most relevant findings were as follows: in men, statistically significant negative correlations were found between SVO_2peak_ and the relative HR expressed as a percentage of HR_peak_ at VT2 (r = −0.252; *p* < 0.05) and VT1 (r = −0.210; *p* < 0.05). In women, however, statistically significant positive correlations were observed between SVO_2peak_ and the relative HR expressed as a percentage of HR_peak_ at VT2 (r = 0.190; *p* < 0.05) and VT1 (r = 0.159; *p* < 0.05).

A similar trend was observed for running speed: in men, SVO_2peak_ showed significant negative correlations with the relative speed expressed as a percentage of SVO_2peak_ at SVT2 (r = −0.197; *p* < 0.05) and SVT1 (r = −0.157; *p* < 0.05). In women, these correlations were positive but low, with SVO_2peak_ and the relative speed expressed as a percentage of SVO_2peak_ at SVT2 (r = 0.117; *p* < 0.05) and SVT1 (r = 0.107; *p* < 0.05).

Significant negative correlations were identified between VO_2peak_ and the relative HR (expressed as a percentage of HR_peak_) at VT2 (r = −0.197; *p* < 0.05) and VT1 (r = −0.169; *p* < 0.05) in men. Conversely, in women, significant positive correlations were detected between VO_2peak_ and the relative HR (expressed as a percentage of HR_peak_) at VT2 (r = 0.196; *p* < 0.05) and VT1 (r = 0.205; *p* < 0.05). Additionally, in men, significant positive correlations were found between SVO_2peak_ and achieving the theoretical maximal HR (r = 0.220; *p* < 0.05). No statistically significant correlations between these variables were observed in the female group.

Based on these results, and with the aim of providing a simpler method for estimating training zones, Table 5 presents the proposed HR training zone estimation for runners, derived as a percentage of SVO_2peak_ and HR_peak_ and categorized according to sex and performance level. Table 6 presents the proposed HR training zone estimation for runners, derived as a percentage of SVT2 and HR at VT2 and categorized according to sex and performance level.

## 4. Discussion

The study objectives were to analyze and present both absolute and relative values of running speed, HR, and VO_2_ at VT2 and VT1 in trained long-distance runners of different performance levels (ranging from beginner to elite) and of both sexes. These data were obtained from progressive incremental exercise tests performed until volitional exhaustion. The overarching goal of this study was to provide reference values to help coaches and athletes more accurately estimate training zones from a progressive exercise test, based on directly measured physiological parameters and considering potential differences related to performance level and sex.

Across the entire sample of 1411 athletes, HRVT2 was reached at 93.5 ± 2.5% of HR_peak_, while HRVT1 occurred at 85.1 ± 4.6% of HR_peak_. When using HRVT2 as the reference value, HR_peak_ represented 107.0 ± 2.9% and HRVT1 90.9 ± 3.7%. Conversely, when SVT2 was taken as the reference point, SVO_2peak_ reached 114.4 ± 5.3%, and SVT1 amounted to 84.4 ± 4.7%. In addition, SVT2 represented 87.6 ± 3.9% of SVO_2peak_, SVT1 corresponded to 73.9 ± 5.5% of SVO_2peak_. These results provide more specific and evidence-based reference ranges derived from a large sample of long-distance runners, offering a more precise and physiologically grounded alternative to the generalized estimates commonly used by commercial training platforms [26]. In addition, the results of the study may help coaches categorize their athletes according to aerobic performance, taking into account variables such as peak speed achieved during an incremental test and years of previous training and competitive experience.

Moreover, the results of the present study partially support the third research hypothesis (i.e., that lower-performance athletes would present higher relative HR values, expressed as a percentage of HR_peak_, at VT1 and VT2 compared with higher-performance athletes). Specifically, in men, both ventilatory thresholds and the HR values at these thresholds, expressed relative to VO_2peak_ and HR_peak_, respectively, were higher in athletes with low aerobic fitness than in those with very high aerobic fitness. This finding contrasts with what would be expected from a physiological standpoint [27]. For example, Benítez et al. Ref. [14] reported that as aerobic performance increases, these relative percentages also tend to be higher. However, that study included participants who were not specialized in running, and the test protocol was individualized according to each subject’s characteristics. In contrast, in the present study, all participants were experienced long-distance runners and performed the same standardized incremental running test (with only the starting speed adjusted to achieve a similar test duration across subjects) contributing to advancing the understanding of training in runners. The most plausible explanation for our results is that lower-level male athletes did not reach their VO_2max_ during the test, stopping before achieving a VO_2_ plateau, thereby artificially elevating their relative values. This interpretation is supported by the correlations found in the present study, where significant negative associations were observed in men between performance, expressed as SVO_2peak_, and the relative HR with respect to HR_peak_ at VT2 (r = −0.252; *p* < 0.05) and VT1 (r = −0.210; *p* < 0.05), as well as with the achievement of the theoretical maximal HR (r = 0.220; *p* < 0.05). Similar results were observed by Anselmi et al. [28] who found that trained athletes showed lower relative values with respect to VO_2peak_ and HR_peak_ than healthy sedentary individuals, possibly because sedentary participants do not reach VO_2max_. This could be explained by differences in durability between groups. In long-distance runners, one of the key variables influencing performance is durability [29]. Lower-level athletes typically exhibit reduced physiological resilience compared to their higher-performing counterparts, which may lead them to experience greater fatigue toward the end of an incremental test. This accumulated fatigue can negatively affect running economy as the test progresses, ultimately preventing them from reaching their true maximal oxygen uptake [17].

On the other hand, the third hypothesis was not supported for relative running speed, as no marked differences were found in SVT2 and SVT1 with respect to SVO_2peak_ across performance levels. This could suggest that men may not reach their VO_2max_ due to biomechanical limitations or muscular fatigue occurring before their physiological limit is achieved. Less experienced and lower-performing runners tend to exhibit greater deterioration in biomechanical variables that influence running economy toward the end of progressive incremental tests [30,31]. This decline may lead to premature termination of the test before reaching their true maximal aerobic power.

However, these trends were not observed in the case of female runners. The results of the present study showed a higher proportion of women reaching their theoretical maximal HR compared with men. Moreover, significant positive correlations were observed between performance, expressed as SVO_2peak_, and the relative HR with respect to HR_peak_ at VT2 and VT1. This may indicate that women are more capable of achieving their true maximal aerobic potential in this type of test across all performance levels.

This could be explained by sex-related differences in substrate utilization during endurance exercise. Women oxidize more lipids and less carbohydrate and protein compared with men, which may result in lower lactate accumulation and greater metabolic resistance to fatigue. Such metabolic efficiency could enable women to sustain higher workloads for longer periods without reaching local or systemic exhaustion before attaining the severe-intensity domain where the VO_2_ plateau occurs [32]. In addition, women appear to exhibit greater resistance to central fatigue, showing smaller reductions in voluntary activation during prolonged moderate-intensity efforts, which could confer an advantage in progressive endurance tests such as the one performed in this study [33]. Consistent with previous research, women also displayed higher relative values of HR, running speed, and VO_2_ at the ventilatory thresholds when expressed as a percentage of HR_peak_, SVO_2peak_, and VO_2peak_, respectively [13,14].

Furthermore, according to the data obtained from the performance tests, considering variables such as sex and performance level when establishing reference parameters becomes essential. In men, HRVT2 represented a higher percentage of HR_peak_ in the lower-level groups (around 93% in low aerobic fitness and low to moderate aerobic fitness runners) compared with very high aerobic fitness runners (approximately 91%). Similarly, HRVT1 was higher in athletes with low aerobic fitness (85% of HRpeak) compared with those with very high aerobic fitness (80% of HRpeak). Women, on the other hand, showed higher relative HR values than men (supporting the first hypothesis of the study) although these remained more stable across performance levels (95% of HR_peak_ at VT2 and 86% at VT1). Regarding running speed, when the peak speed obtained from a progressive test is used as reference, the relative values corresponding to SVT2 and SVT1 were similar across performance levels (87% and 73%, respectively, in men; and 89% and 75% for SVT2 and SVT1, respectively, in women). This results also support the second hypothesis of the study showing higher relative running speed values (as a percentage of maximal speed reached at the test) at the ventilatory thresholds compared with male runners. Therefore, considering these data and the physiological differences that may influence the results, both performance level and sex must be taken into account when estimating exercise intensities from a progressive test in runners—particularly when using HR as the main variable to monitor intensity [13,14].

Therefore, caution should be exercised when prescribing training intensities based on general criteria without considering key factors such as sex, performance level, and the type of test used to estimate training zones (e.g., progressive vs. constant-load protocols). The increasing reliance on automated training software and wearable-based platforms has led to a growing lack of individualization in training prescription [18]. These systems often neglect to account for physiological and biomechanical differences between athletes, which may result in a mismatch between the intended and the actual metabolic state being targeted during training. Furthermore, many of these applications tend to overlook specific work on physiological milestones relevant to endurance performance, prioritizing simplicity and scalability over accuracy, particularly for coaches managing large athlete volumes. Consequently, individualized assessment and prescription remain essential to ensure optimal performance development and safeguard athlete health.

Although the reference ranges proposed in this study provide a more precise and evidence-based alternative to the generalized estimates offered by commercial platforms, individual variability remains an essential consideration. Even with improved standards, some athletes naturally fall outside the expected ranges. In this context, the increasing accessibility of metabolic testing represents a valuable tool for both athletes and coaches. Beyond helping to refine training zone estimation, these assessments provide key insights into performance determinants such as VO_2max_, RE, and substrate utilization profiles (e.g., Fatmax, carbohydrate oxidation rates across intensity domains), allowing for a more comprehensive and individualized approach to endurance training prescription [34].

This study makes a valuable contribution to the field by establishing new standards for estimating training zones in runners, owing to its larger sample size, while also demonstrating how the estimation of training intensities from progressive exercise tests can be influenced by both performance level and sex. However, several limitations should be considered when interpreting these results. One of the main limitations of this study is the smaller number of female participants classified as having high and very high aerobic fitness compared with their male counterparts, which may have reduced the statistical power to detect differences between performance levels in women. Additionally, the classification of athletes based solely on VO_2peak_ values might not fully capture performance differences specific to running, as this parameter does not account for biomechanical efficiency, durability, or running economy. Another limitation is that the number of years of structured training experience was not considered in the analysis, which could have influenced the physiological responses and relative intensity values observed across groups.

## 5. Conclusions

The present study provides valuable reference data for estimating training intensities in long-distance runners based on progressive incremental tests, highlighting the influence of both performance level and sex. In male runners, lower-level athletes exhibited higher relative HR values at the ventilatory thresholds compared with elite athletes, likely due to not reaching their maximal aerobic performance in the test. In contrast, female runners could be more consistently able to achieve their maximal aerobic potential, showing higher and more stable relative HR and running speed values across all performance levels. These findings underscore the importance of considering individual physiological characteristics when prescribing exercise intensities and planning training, rather than relying on generalized percentages that do not account for sex, performance level or test type. Ultimately, individualized assessment and prescription remain critical to optimizing performance development, targeting specific physiological milestones, and maintaining athlete health in long-distance running.

## Figures and Tables

**Table 1:** Physiological variables obtained from the performance tests according to performance level and sex.

	Very High Aerobic Fitness	High Aerobic Fitness	Moderately Aerobic Fitness	Low to Moderate Aerobic Fitness	Low Aerobic Fitness
Male	n = 22	n = 35	n = 156	n = 258	n = 348
VO_2peak_ (mL·kg^−1^·min^−1^)	79.2 ± 4.4	72.8 ± 1.3	65.9 ± 2.5	57.8 ± 2.2	46.6 ± 5.5
Years of experience (n)	9.7 ± 1.6	8.1 ± 2.6	6.1 ± 2.5	4.2 ± 2.0	3.1 ± 1.7
SVO_2peak_ (km/h)	22.0 ± 1.1	20.9 ± 1.4	18.6 ± 1.9	16.4 ± 1.4	14.0 ± 1.8
Economy (mL·kg^−1^·min^−1^)	194.5 ± 16.4	199.0 ± 22.6	207.1 ± 27.2	218.0 ± 25.2	219.7 ± 25.8
SVT2 (km/h)	19.2 ± 0.6	18.1 ± 1.4	16.1 ± 1.7	14.2 ± 1.4	12.1 ± 1.6
SVT1 (km/h)	15.5 ± 0.7	15.3 ± 1.0	13.6 ± 1.3	11.9 ± 1.2	10.2 ± 1.5
%VO_2peak_ VT2 (%)	83.8 ± 5.1	85.1 ± 4.8	85.9 ± 5.6	87.1 ± 5.9	87.4 ± 5.3
%VO_2peak_ VT1 (%)	67.5 ± 5.9	69.1 ± 6.4	70.1 ± 8.5	73.2 ± 6.9	73.4 ± 8.3
THRmax (%)	83.6	77.4	73.1	65.3	63.2
Female	n = 6	n = 16	n = 56	n = 105	n = 409
VO_2max_ (mL·kg^−1^·min^−1^)	68.4 ± 1.8	63.3 ± 1.4	58.0 ± 1.7	52.4 ± 1.7	40.9 ± 6.1
Years of experience (n)	8.2 ± 1.3	7.3 ± 2.4	5.4 ± 2.3	3.8 ± 1.7	2.6 ± 1.4
Economy (mL·kg^−1^·min^−1^)	203.4 ± 23.6	204.5 ± 24.9	215.2 ± 23.8	225.9 ± 25.3	223.6 ± 24.9
SVO_2peak_ (km/h)	17.7 ± 0.6	17.7 ± 1.2	16.3 ± 1.2	14.5 ± 1.3	12.2 ± 1.9
SVT2 (km/h)	15.8 ± 0.4	15.6 ± 0.9	14.4 ± 0.9	12.9 ± 1.1	10.7 ± 1.7
SVT1 (km/h)	13.6 ± 0.5	13.3 ± 0.9	12.6 ± 1.0	11.1 ± 1.2	9.1 ± 1.6
%VO_2peak_ VT2	84.5 ± 5.6	88.4 ± 3.6	89.0 ± 4.9	88.0 ± 5.4	88.1 ± 6.9
%VO_2peak_ VT1	69.2 ± 7.5	72.7 ± 5.3	77.3 ± 7.0	75.3 ± 7.7	75.1 ± 9.3
THRmax (%)	83.3	81.3	74.4	71.1	71.9

Note: VO_2peak_ = Peak value of oxygen consumption; mL = millilitres; Kg = kilograms; min = minute; SVO_2peak_ = Speed corresponding to peak oxygen consumption; km = kilometre; h = Hour; SVT2 = Speed corresponding to second ventilatory threshold; SVT1 = Speed corresponding to first ventilatory threshold; %VO_2peak_ VT2 = Percentage of oxygen consumption relative to peak oxygen consumption at second ventilatory threshold; %VO_2peak_ VT1 = Percentage of oxygen consumption relative to peak oxygen consumption at first ventilatory threshold; VO_2max_ Plateau = Percentage of the sample that reached the plateau in oxygen consumption; THR_max_ = Percentage of the sample that reached the theoretical maximum heart rate.

**Table 2:** Relative heart rate and running speed according to performance level and sex.

	Global	Very High Aerobic Fitness	High Aerobic Fitness	Moderately Aerobic Fitness	Low to Moderate Aerobic Fitness	Low Aerobic Fitness
**Male**	n = 819	n = 22	n = 35	n = 156	n = 258	n = 348
**%HRpeak**						
VT2 (%)	93.0 ± 2.5 *	90.8 ± 2.1 *	91.9 ± 2.4 *	92.7 ± 2.5 *	93.0 ± 2.5 *	93.4 ± 2.4 *
VT1(%)	84.2 ± 4.7 *	80.5 ± 3.9 *	81.8 ± 4.7 *	83.3 ± 5.2 *	84.5 ± 4.2 *	84.8 ± 4.6 *
**%VT2. HR**						
VO_2peak_ (%)	107.6 ± 2.6 *	110.2 ± 2.6 *	108.7 ± 2.9	107.9 ± 2.3 *	107.6 ± 2.9 *	107.1 ± 2.8 *
VT1(%)	86.9 ± 3.9 *	88.6 ± 4.6	88.9 ± 3.9 *	89.8 ± 3.9 *	90.8 ± 3.5 *	90.7 ± 3.7 *
**%SVO_2peak_**						
VT2 (%)	86.9 ± 3.9 *	86.5 ± 3.6	86.7 ± 3.7	86.8 ± 3.9 *	86.7 ± 3.8 *	87.1 ± 4.0 *
VT1(%)	73.1 ± 5.1 *	72.8 ± 3.8 *	73.5 ± 3.9	73.1 ± 5.0 *	73.2 ± 4.7 *	72.9 ± 5.6 *
**%SVT2S**						
VO_2peak_ (%)	115.3 ± 5.3 *	115.8 ± 4.9	115.6 ± 5.1	115.4 ± 5.4 *	115.6 ± 5.2 *	115.0 ± 5.4 *
VT1 (%)	84.1 ± 4.5 *	84.1 ± 3.4 *	84.8 ± 3.4	84.2 ± 4.6 *	84.4 ± 4.2 *	83.7 ± 4.8
**Female**	n = 592	n = 6	n = 16	n = 56	n = 105	n = 409
**%HRpeak**						
VT2 (%)	94.2 ± 2.2 *	94.7 ± 1.9 *	93.9 ± 1.9 *	94.9 ± 2.0 *	94.7 ± 2.2 *	94.0 ± 2.3 *
VT1 (%)	86.2 ± 4.3 *	86.2 ± 3.6 *	85.9 ± 4.9 *	87.4 ± 3.6 *	87.3 ± 3.6 *	86.0 ± 4.5 *
**%VT2. HR**						
VO_2peak_ (%)	106.2 ± 2.6 *	105.6 ± 2.6 *	106.5 ± 2.2	105.4 ± 2.2 *	105.6 ± 2.5 *	106.4 ± 2.6 *
VT1 (%)	91.6 ± 3.5 *	91.1 ± 2.8	91.5 ± 4.1 *	92.0 ± 2.8 *	92.2 ± 2.8 *	91.3 ± 3.7 *
**%SVO_2peak_**						
VT2 (%)	88.6 ± 3.8 *	89.4 ± 3.7	87.9 ± 3.4	88.9 ± 3.4 *	89.5 ± 3.2 *	88.4 ± 3.9 *
VT1 (%)	75.2 ± 5.8 *	76.8 ± 2.9 *	75.2 ± 3.6	77.5 ± 3.3 *	76.7 ± 4.9 *	74.5 ± 6.2 *
**%SVT2**						
VO_2peak_ (%)	113.0 ± 4.9 *	112.0 ± 4.8	113.9 ± 4.4	112.6 ± 4.3 *	111.9 ± 4.1 *	113.3 ± 5.2 *
VT1 (%)	84.8 ± 4.9 *	86.0 ± 3.0 *	85.7 ± 3.0	87.2 ± 2.9 *	85.7 ± 4.3 *	84.2 ± 5.1

Note: * = Statistically significative difference between sex; %HR_peak_ = Relative heart rate values as percentage of the peak heart rate; VT2 = Second ventilatory threshold; VT1 = First ventilatory threshold; VO_2peak_ = Peak value of oxygen consumption; %VT2. HR = Relative heart rate values as percentage of the heart rate at second ventilatory threshold; %SVO_2peak_ = Relative speed value as a percentage of the speed at peak value of oxygen consumption; %SVT2 = Relative speed value as a percentage of the speed at second ventilatory threshold.

**Table 3:** Differences in relative heart rate at second ventilatory threshold as percentage of heart rate peak between groups: statistical significance and Cohen’s d effect size.

			Very High Aerobic Fitness	High Aerobic Fitness	Moderately Aerobic Fitness	Low to Moderate Aerobic Fitness
Males	High aerobic fitness	*p*	0.066			
		ES	−0.51			
	Moderately aerobic fitness	*p*	0.001	0.128		
		ES	−0.78	−0.29		
	Low to moderate aerobic fitness	*p*	<0.001	0.027	0.238	
		ES	−0.88	−0.4	−0.12	
	Low aerobic fitness	*p*	<0.001	0.001	0.002	0.03
		ES	−1.05	−0.6	−0.3	−0.18
Females	High aerobic fitness	*p*	0.391			
		ES	0.42			
	Moderately aerobic fitness	*p*	0.823	0.079		
		ES	−0.10	−0.5		
	Low to moderate aerobic fitness	*p*	0.974	0.154	0.651	
		ES	−0.14	−0.38	0.08	
	Low aerobic fitness	*p*	0.453	0.844	0.005	0.003
		ES	0.31	−0.05	0.4	0.32

Note: *p* = Statistical significance; ES: Effect size.

**Table 4:** Differences in relative heart rate first ventilatory threshold as percentage of heart rate peak between groups: statistical significance and Cohen’s d effect size.

			Very High Aerobic Fitness	High Aerobic Fitness	Moderately Aerobic Fitness	Low to Moderate Aerobic Fitness
Males	High aerobic fitness	*p*	0.266			
		ES	−0.31			
	Moderately aerobic fitness	*p*	0.014	0.120		
		ES	−0.57	−0.29		
	Low to moderate aerobic fitness	*p*	<0.001	0.001	0.015	
		ES	−0.96	−0.63	−0.40	
	Low aerobic fitness	*p*	<0.001	<0.001	0.002	0.508
		ES	−0.94	−0.63	−0.30	−0.05
Females	High aerobic fitness	*p*	0.905			
		ES	0.06			
	Moderately aerobic fitness	*p*	0.465	0.210		
		ES	−0.32	−0.36		
	Low to moderate aerobic fitness	*p*	0.465	0.178	0.949	
		ES	−0.31	−0.29	0.11	
	Low aerobic fitness	*p*	0.844	0.935	0.016	0.002
		ES	0.08	0.02	0.34	0.34

Note: *p* = Statistical significance; ES: Effect size.

**Table 5:** Proposed heart rate and speed training zones estimation in runners based on the maximal speed achieved during progressive exercise test, according to sex and performance level.

		Very High Aerobic Fitness	High Aerobic Fitness	Moderately Aerobic Fitness	Low to Moderate Aerobic Fitness	Low Aerobic Fitness
**Male**	**Speed Zones**					
	SVO_2peak_ (%)	99–101	99–101	99–101	99–101	99–101
	SVT2 (%)	86–88	86–88	86–88	86–88	86–88
	SVT1 (%)	72–74	72–74	72–74	72–74	72–74
	**Heart rate zones**					
	HRpeak (%)	99–101	99–101	99–101	99–101	99–101
	HRVT2 (%)	90–92	91–93	91.5–93.5	92–94	92.5–94.5
	HRVT1 (%)	79–81	81–83	82–84	83.5–85.5	84–86
**Female**	**Speed Zones**					
	SVO_2peak_ (%)	99–101	99–101	99–101	99–101	99–101
	SVT2 (%)	88–90	87–89	88–90	88–90	87–89
	SVT1(%)	76–78	74–76	76–78	76–78	74–76
	**Heart rate zones**					
	HRpeak (%)	99–101	99–101	99–101	99–101	99–101
	HRVT2 (%)	94–96	93–95	94–96	94–96	93–95
	HRVT1 (%)	85–87	85–87	86–88	86–88	85–87

Note: SVO_2peak_ (%) = Relative speed value as a percentage of the speed at peak value of oxygen consumption; SVT2 (%) = Relative speed value as a percentage of the speed at peak value of oxygen consumption at second ventilatory threshold; SVT1 (%) = Relative speed value as a percentage of the speed at peak value of oxygen consumption at first ventilatory threshold; HRpeak (%) = Relative heart rate values as percentage of the peak heart rate; HRVT2 (%) = Relative heart rate values as percentage of the peak heart rate at second ventilatory threshold; HRVT1 (%) = Relative heart rate values as percentage of the peak heart rate at first ventilatory threshold.

**Table 6:** Proposed heart rate and speed training zones estimation in runners based on the speed at the ventilatory threshold 2 during progressive exercise test, according to sex and performance level.

		Very High Aerobic Fitness	High Aerobic Fitness	Moderately Aerobic Fitness	Low to Moderate Aerobic Fitness	Low Aerobic Fitness
**Male**	**Speed Zones**					
	SVO_2peak_ (%)	114–116	115–117	114.5–116.5	114.5–116.5	114.5–116.5
	SVT2 (%)	99–101	99–101	99–101	99–101	99–101
	SVT1 (%)	83–85	84–86	83–85	83.5–85.5	83–85
	**Heart rate zones**					
	HRpeak (%)	109–111	108–110	107–109	106.5–108.5	106–108
	HRVT2 (%)	99–101	99–101	99–101	99–101	99–101
	HRVT1 (%)	86–89	87.5–89.5	88–90	89–91	90–92
**Female**	**Speed Zones**					
	SVO_2peak_ (%)	111–113	113–115	111.5–113.5	111–113	112–114
	SVT2 (%)	99–101	99–101	99–101	99–101	99–101
	SVT1(%)	85–87	85–87	86–88	85–87	83–85
	**Heart rate zones**					
	HRpeak (%)	104.5–106.5	105.5–107.5	104.5–106.5	104.5–106.5	105.5–107.5
	HRVT2 (%)	99–101	99–101	99–101	99–101	99–101
	HRVT1 (%)	90–92	90.5–92.5	91–93	91–93	91–93

Note: SVO_2peak_ (%) = Relative speed value as a percentage of the speed at peak value of oxygen consumption; SVT2 (%) = Relative speed value as a percentage of the speed at peak value of oxygen consumption at second ventilatory threshold; SVT1 (%) = Relative speed value as a percentage of the speed at peak value of oxygen consumption at first ventilatory threshold; HRpeak (%) = Relative heart rate values as percentage of the peak heart rate; HRVT2 (%) = Relative heart rate values as percentage of the peak heart rate at second ventilatory threshold; HRVT1 (%) = Relative heart rate values as percentage of the peak heart rate at first ventilatory threshold.

## Data Availability

The data presented in this study are available on request from the corresponding author due to privacy and ethical restrictions (specify the reason for the restriction).
